# Subtype-specific causal effects of hypothyroidism on obstructive sleep apnea: A bidirectional Mendelian randomization study

**DOI:** 10.1097/MD.0000000000043266

**Published:** 2025-07-04

**Authors:** Hao Zhang, Zhimin Wu, Qiu Chen, Guodong Yu, Liang Chen, Yifei Ma, Yi Chen

**Affiliations:** a Department of Anesthesiology, The Second People’s Hospital of Hefei, Hefei Hospital Affiliated to Anhui Medical University, Hefei, Anhui, China; b Department of Otorhinolaryngology – Head and Neck Surgery, The Maternal and Child Health Care Hospital of Guizhou Medical University, Guiyang, Guizhou, China; c Department of Otorhinolaryngology – Head and Neck Surgery, Affiliated Hospital of Guizhou Medical University, Guiyang, Guiyang, Guizhou, China; d Cancer Research Institute, the Affiliated Cancer Hospital of Xinjiang Medical University, Urumqi, Xinjiang Uygur Autonomous Region, China.

**Keywords:** causal inference, hypothyroidism, Mendelian randomization, obstructive sleep apnea, subtype analysis, thyroglobulin

## Abstract

This study investigates the subtype-specific causal relationships between hypothyroidism (HT) and obstructive sleep apnea (OSA) using a bidirectional Mendelian randomization (MR) design. A two-sample MR analysis was conducted based on genome-wide association study summary statistics from European populations, including multiple HT subtypes and thyroid function indicators. The inverse variance weighted method was used as the primary analysis, supplemented by MR-Egger regression, weighted median, and leave-one-out analyses. Sensitivity analyses assessed pleiotropy and heterogeneity. Bidirectional MR was performed to explore causal directions between HT and OSA. Hypothyroidism, drug reimbursement, hypothyroidism, strict autoimmune, and congenital iodine deficiency syndrome/HT were significantly associated with an increased risk of OSA. Thyroglobulin exhibited a protective causal effect against OSA. In the reverse MR analysis, genetically predicted OSA was found to causally increase the risk of hypothyroidism, drug reimbursement. No substantial pleiotropy was detected, and the findings were robust across multiple analytical methods. This study revealed subtype-specific and bidirectional causal associations between HT and OSA. The findings highlight the need for personalized screening and management strategies for patients with different forms of thyroid dysfunction.

**Key points**

- An association exists between hypothyroidism and OSA, but traditional observational studies are limited by confounding factors and reverse causality, making it difficult to establish true causal effects.
- This study is the first to reveal differential causal effects of different subtypes of hypothyroidism on OSA and identify thyroglobulin as a potentially protective factor against OSA.
- Clinicians should assess and screen OSA risk in patients with drug-reimbursement and autoimmune hypothyroidism.
- The results provide a theoretical foundation for personalized diagnostic and therapeutic strategies.

## 1. Introduction

Obstructive sleep apnea (OSA) is a widely prevalent sleep disorder characterized by recurrent obstruction of the upper airway, leading to intermittent drops in blood oxygen saturations.^[1]^ According to data from The Lancet in 2021, approximately 1 billion individuals aged 30 to 69 worldwide are affected by OSA.^[2]^ The accompanying symptoms, such as daytime sleepiness and concentration difficulties, not only significantly reduce quality of life but may also precipitate various complications, including stroke, diabetes, and hypertension.^[3,4]^ In addition, growing evidence has suggested a potential association between OSA and tumorigenesis, particularly in hormone-sensitive cancers such as breast cancer, possibly due to chronic intermittent hypoxia and systemic inflammation.^[5–8]^ Hypothyroidism (HT), a common metabolic disorder, has a prevalence rate of 0.3% to 3.7% in the U.S. population and 0.2% to 5.3% in Europe.^[9]^ Clinical data indicate that around 40% of hypothyroid patients also have OSA,^[10]^ and there is a positive correlation between decreased thyroid hormone levels and the severity of OSA^[11]^ Although thyroid replacement therapy has been shown to improve OSA symptoms,^[12]^ the causal relationship between the 2 remains controversial. Some studies have found that HT significantly increases the risk of developing OSA,^[13]^ whereas others have not observed significant abnormalities in thyroid function among OSA patients.^[14]^ These inconsistencies may be attributed to selection biases, publication biases, and uncontrolled confounding factors in study designs.

Recent Mendelian randomization (MR) studies have provided new perspectives for elucidating the causal relationship between the 2 conditions. MR analyses by Zhao et al and Lu et al confirmed a significant association between genetically predicted HT and the risk of OSA.^[15–17]^ However, these studies did not adequately consider the heterogeneity of HT subtypes (including autoimmune, iodine deficiency syndrome, drug-induced, and postinfectious variants). Previous research has demonstrated that different subtypes exhibit significant clinical heterogeneity; for instance, autoimmune HT is closely related to depression, whereas iodine deficiency and postinfectious variants do not share this association. Such subtype-specific effects may also exist in the interaction between HT and OSA. Notably, research on the impact of OSA on thyroid function is relatively scarce, although evidence suggests that OSA-related intermittent hypoxia may affect thyroid function. Furthermore, existing studies have yet to systematically integrate an analysis of thyroid-related biomarkers and subtype-specific effects.

This study employs a bidirectional Mendelian randomization approach to integrate data on thyroid-related biomarkers, systematically assessing the differential causal effects of various subtypes of HT on OSA. By combining genome-wide association study (GWAS) data from the IEU database and biobank data from FinnGen, we delineate key subtypes of HT and analyze their specific impacts on the risk of OSA, aiming to provide a more reliable theoretical basis for clinical precision screening and individualized intervention.

## 2. Methods

### 2.1. Study design and data sources

This study employed a bidirectional MR design to investigate the causal associations between HT and its subtypes and OSA. We integrated information from 2 primary data sources: (1) genomic data on thyroid-related biomarkers from the IEU Open GWAS database; and (2) genetic information regarding HT subtypes and OSA from the Finnish FinnGen biobank. The FinnGen project includes genomic and health record data from approximately 500,000 individuals in the Finnish population, providing high-quality and representative information on disease-related genetic variations. This study utilized only summary statistics and did not involve any personal-level privacy information.

### 2.2. Definition of exposure and outcome

In this study, HT and its main subtypes were considered as exposure variables, while OSA was treated as the outcome variable (Table 1). Based on clinical diagnoses and treatment records in the FinnGen database, HT was further subdivided into the following subtypes: (1) hypothyroidism, drug reimbursement (HT-DR): defined as clinically confirmed HT recorded for the purpose of medication reimbursement, typically reflecting long-term disease requiring hormone replacement therapy. (2) Hypothyroidism, strict autoimmune (HT-SA): represents autoimmune HT, such as Hashimoto thyroiditis, confirmed by diagnostic records and the presence of thyroid-specific autoantibodies. (3) Congenital iodine-deficiency syndrome/HT (CIDS): describes congenital HT primarily resulting from insufficient iodine intake during pregnancy, often associated with impaired neurodevelopment and abnormal thyroid morphology. (4) Postinfectious hypothyroidism (PIH): refers to HT occurring after infectious illnesses, potentially due to subacute thyroiditis or immune-mediated damage to the thyroid gland. (5) Hypothyroidism due to medicaments and other exogenous substances (HT-ME): encompasses HT caused by drugs (e.g., lithium, amiodarone) or external substances such as iodine-containing compounds. Additionally, to comprehensively analyze the potential effects of thyroid function factors on OSA, the study sourced key biomarker data related to thyroid function from the IEU Open GWAS database, including thyroid-stimulating hormone (TSH), thyrotropin-releasing hormone (TRH), and thyroglobulin (Tg).

**Table 1:** Summary of genetic data information regarding exposure and outcome phenotypes.

Trait	Consortium	Sample	Population	GWAS ID
Hypothyroidism, drug reimbursement (HT-DR)	FinnGen	119151	European	finngen_R11_HYPOTHY_REIMB
Hypothyroidism, strict autoimmune (HT-SA)	FinnGen	378830	European	finngen_R11_E4_HYTHY_AI_DX
Postinfectious hypothyroidism (PIH)	FinnGen	385815	European	finngen_R11_E4_HYTHYPOSTINF
Hypothyroidism due to medicaments and other exogenous substances (HT-ME)	FinnGen	385952	European	finngen_R11_E4_HYTHYSUBS
Congenital iodine-deficiency syndrome/hypothyroidism (CIDS)	FinnGen	386636	European	finngen_R11_E4_CONGEIOD
Thyrotropin-releasing hormone (TRH)	MRC-IEU	3310	European	prot-a-530
Thyroid stimulating hormone (TSH)	MRC-IEU	3310	European	prot-a-3102
Thyroglobulin (Tg)	MRC-IEU	3310	European	prot-a-2960
Obstructive sleep apnea (OSA)	FinnGen	451684	European	finngen_R11_G6_SLEEPAPNO

GWAS = genome-wide association study.

Although both the exposure and outcome datasets were derived from the FinnGen biobank, they were based on independent GWAS conducted on different phenotypes, minimizing the risk of substantial sample overlap. Additionally, all selected instrumental variables had *F*-statistics >10, indicating strong instrument strength and reducing the likelihood of weak instrument bias or overlap-related bias.

### 2.3. Mendelian randomization assumptions

Mendelian randomization assumptions: this MR study was based on 3 core assumptions. First, the relevance assumption, which requires that the selected genetic variants (instrumental variables) are strongly associated with the exposure of interest. Second, the independence assumption, meaning that the genetic variants are independent of any confounders that could bias the exposure–outcome association. Third, the exclusion restriction assumption, which states that the genetic variants affect the outcome exclusively through the exposure, not through alternative pathways.^[18]^

### 2.4. Selection of instrumental variables

For each exposure variable (including the aforementioned subtypes of HT and biomarkers such as TSH, TRH, and Tg), we selected candidate genetic instrumental variables based on the following criteria^[18]^: (1) significant correlation with the exposure (*P* < 5 × 10^−8^; if fewer than 3 single nucleotide polymorphisms (SNPs) were available, the threshold was relaxed to *P* < 5 × 10^−6^), (2) absence of strong linkage disequilibrium (*r*² < 0.001, distance > 10,000 kb), and (3) absence of apparent pleiotropy (assessed through MR-Egger regression). To further minimize potential confounding, we manually verified each selected SNP using the PhenoScanner database (https://ldlink.nih.gov/?tab=home). SNPs found to be associated with known confounders, such as obesity, smoking, alcohol consumption, or other sleep disorders, were excluded from the analysis. All selected SNPs were harmonized between the exposure and outcome datasets to ensure consistent effect allele orientation. If palindromic SNPs with intermediate allele frequencies were encountered, they were removed to avoid ambiguity.

### 2.5. Statistical analysis

We primarily used the inverse variance weighted (IVW) method to estimate causal effects, simultaneously employing the weighted median method and MR-Egger regression to test the robustness of the results. To evaluate the bidirectional causal associations between the 2 diseases, we conducted reverse MR analysis, treating OSA as the exposure and each subtype of HT as the outcome. All analyses were performed in the R software (version 4.0.3) environment, utilizing packages such as “TwoSampleMR.”

### 2.6. Sensitivity analysis

To ensure the reliability of the results, we conducted 3 types of sensitivity analyses: (1) heterogeneity tests (Cochran Q statistic) to assess the consistency of the effects of instrumental variables; (2) horizontal pleiotropy tests (MR-Egger intercept) to evaluate whether there are genetic variations directly influencing the outcome; and (3) leave-one-out analysis to assess the stability of results by sequentially excluding individual SNPs. In all tests, a *P*-value of <.05 was considered statistically significant.

## 3. Results

### 3.1. Causal effects of HT on OSA (positive MR analysis)

**Figure 1.**
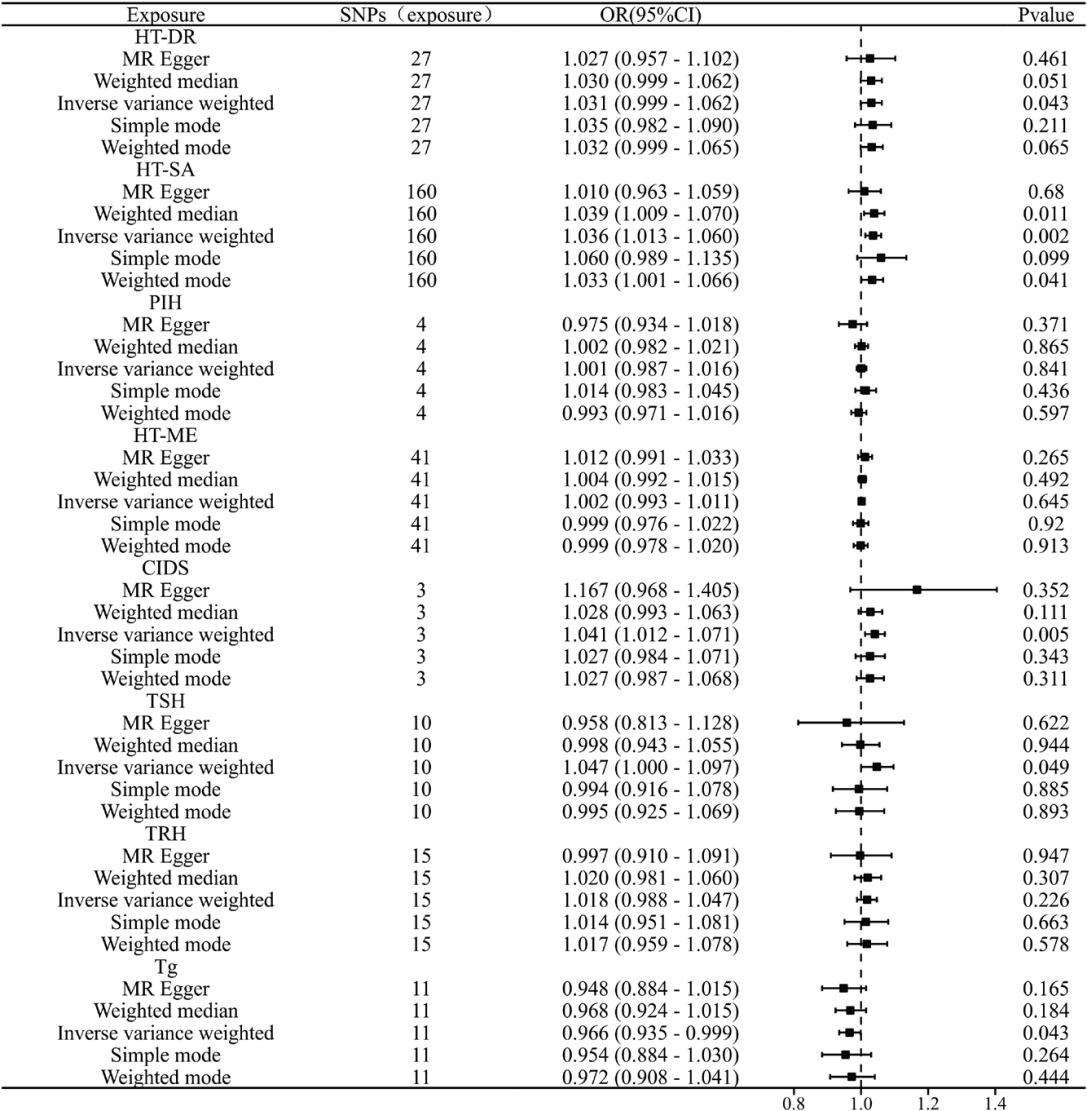
MR analysis results of hypothyroidism subtypes and thyroid function biomarkers associated with OSA. MR = Mendelian randomization.

**Figure 2.**
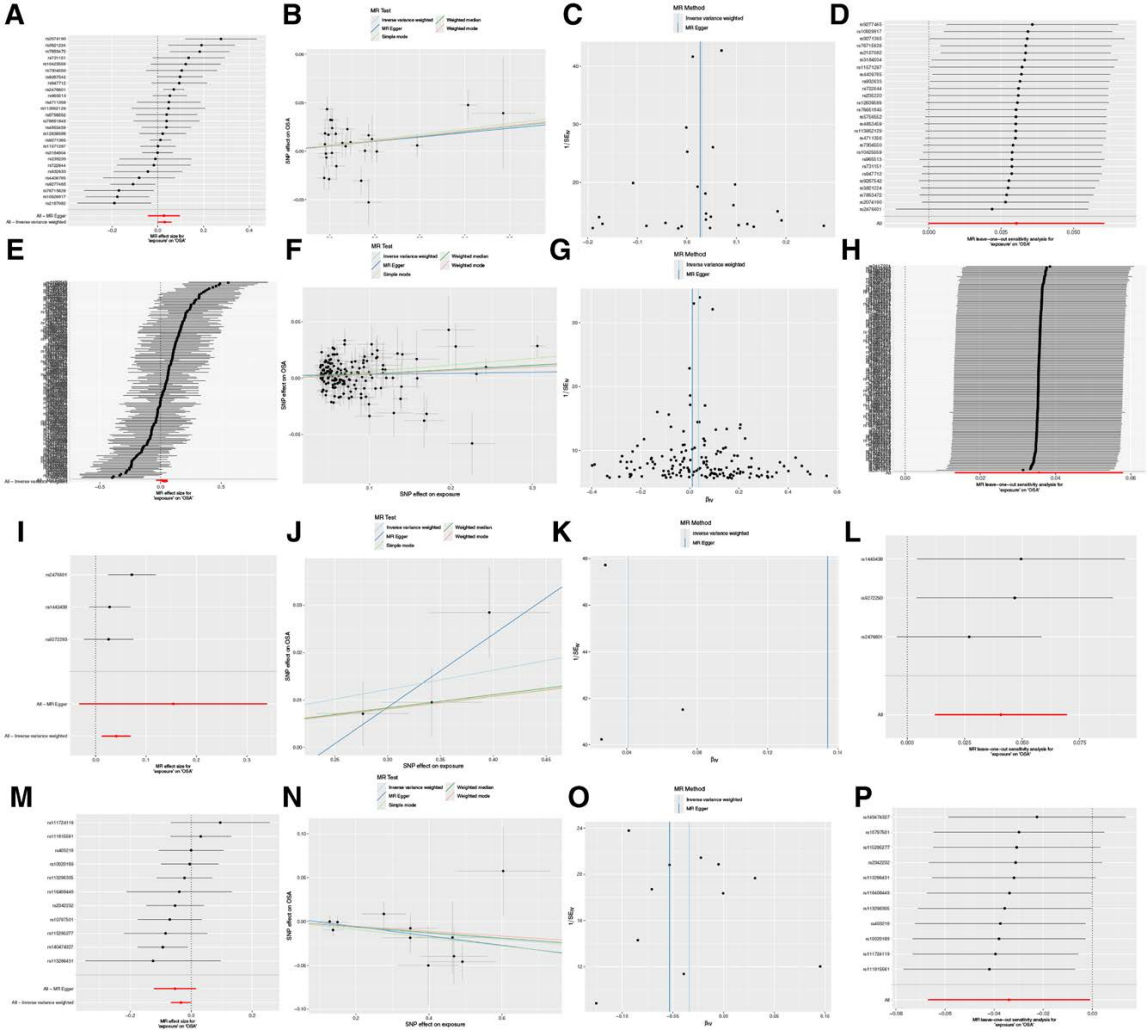
Mendelian randomization results for the causal association between specific hypothyroidism subtypes and OSA risk. (A–D) Causal relationship between HT-DR and OSA: Panel A shows the forest plot; Panel B shows the scatter plot; Panel C shows the funnel plot; Panel D shows the leave-one-out sensitivity analysis plot. (E–H) Causal relationship between HT-SA and OSA: Panel E shows the forest plot; Panel F shows the scatter plot; Panel G shows the funnel plot; Panel H shows the leave-one-out sensitivity analysis plot. (I–L) Causal relationship between CIDS and OSA: Panel I shows the forest plot; Panel J shows the scatter plot; Panel K shows the funnel plot; Panel L shows the leave-one-out sensitivity analysis plot. (M–P) Causal relationship between Tg and OSA: Panel M shows the forest plot; Panel N shows the scatter plot; Panel O shows the funnel plot; Panel P shows the leave-one-out sensitivity analysis plot. CIDS = congenital iodine deficiency syndrome/hypothyroidism, HT-DR: hypothyroidism, drug reimbursement, HT-SA = hypothyroidism, strict autoimmune, OSA = obstructive sleep apnea, Tg = thyroglobulin.

Through MR analysis, we evaluated the causal effects of HT on OSA, with detailed results presented in Fig. 1. Additionally, the positive results of our analyses are showcased in Fig. 2 (refer to the legends for specifics). The findings indicate a significant positive causal relationship between HT-DR and OSA, with an IVW odds ratio (OR) of 1.031 (95% confidence interval [CI]: 1.001–1.062, *P* = .043). This finding was corroborated by the weighted median method, MR-Egger regression, and other approaches. Furthermore, HT-SA also exhibited a significant association with OSA, yielding an IVW OR of 1.036 (95% CI: 1.013–1.060, *P* = .002). CIDS displayed a positive causal effect as well, with an IVW OR of 1.041 (95% CI: 1.012–1.071, *P* = .005).

In contrast, PIH and HT-ME did not show a significant causal relationship with OSA, as demonstrated by the IVW results (PIH: OR = 1.001; 95% CI: 0.987–1.016; *P* = .841; HT-ME: OR = 1.002; 95% CI: 0.993–1.011; *P* = .645). Therefore, we conclude that there is no conclusive causal relationship between these 2 subtypes and OSA.

Regarding thyroid biomarkers, TSH and TRH did not show significant causal effects on OSA (*P* > .05). Notably, Tg exhibited a significant negative causal effect on OSA, with an IVW OR of 0.966 (95% CI: 0.935–0.999; *P* = .043), and this finding was consistent across all methods (*P* < .05).

### 3.2. Causal effects of OSA on HT (reverse MR analysis)

**Figure 3.**
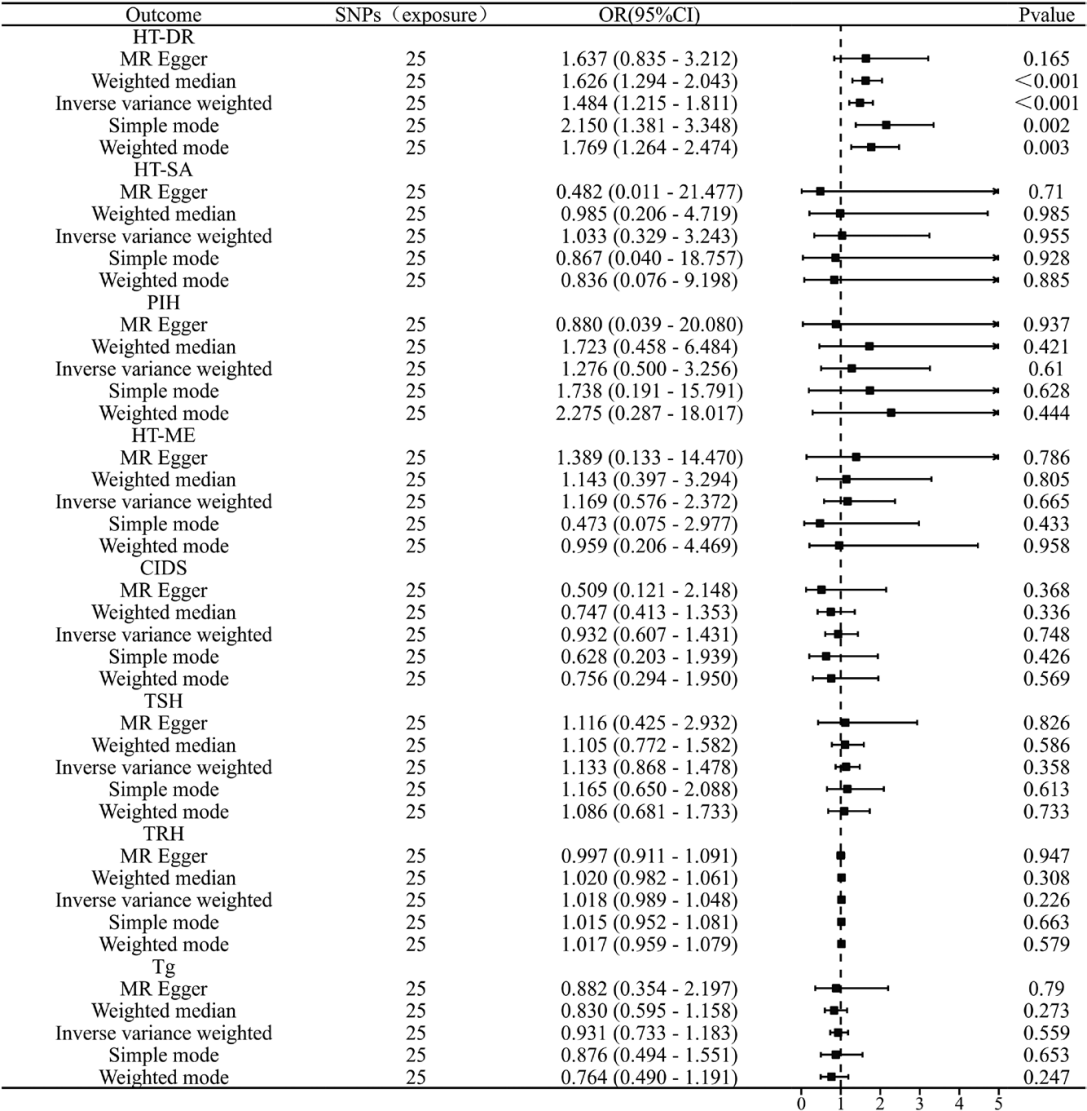
Reverse MR analysis results of OSA with hypothyroidism subtypes and thyroid function biomarkers. OSA = obstructive sleep apnea.

In the reverse MR analysis (Fig. 3), the impact of OSA on most HT subtypes was not significant, including HT-ME (IVW OR = 1.169, 95% CI: 0.576–2.372, *P* = .665), PIH (IVW OR = 1.276, 95% CI: 0.500–3.256, *P* = .610), HT-SA (IVW OR = 1.033, 95% CI: 0.329–3.243, *P* = .955), and CIDS (IVW OR = 0.932, 95% CI: 0.607–1.431, *P* = .748).

**Figure 4.**
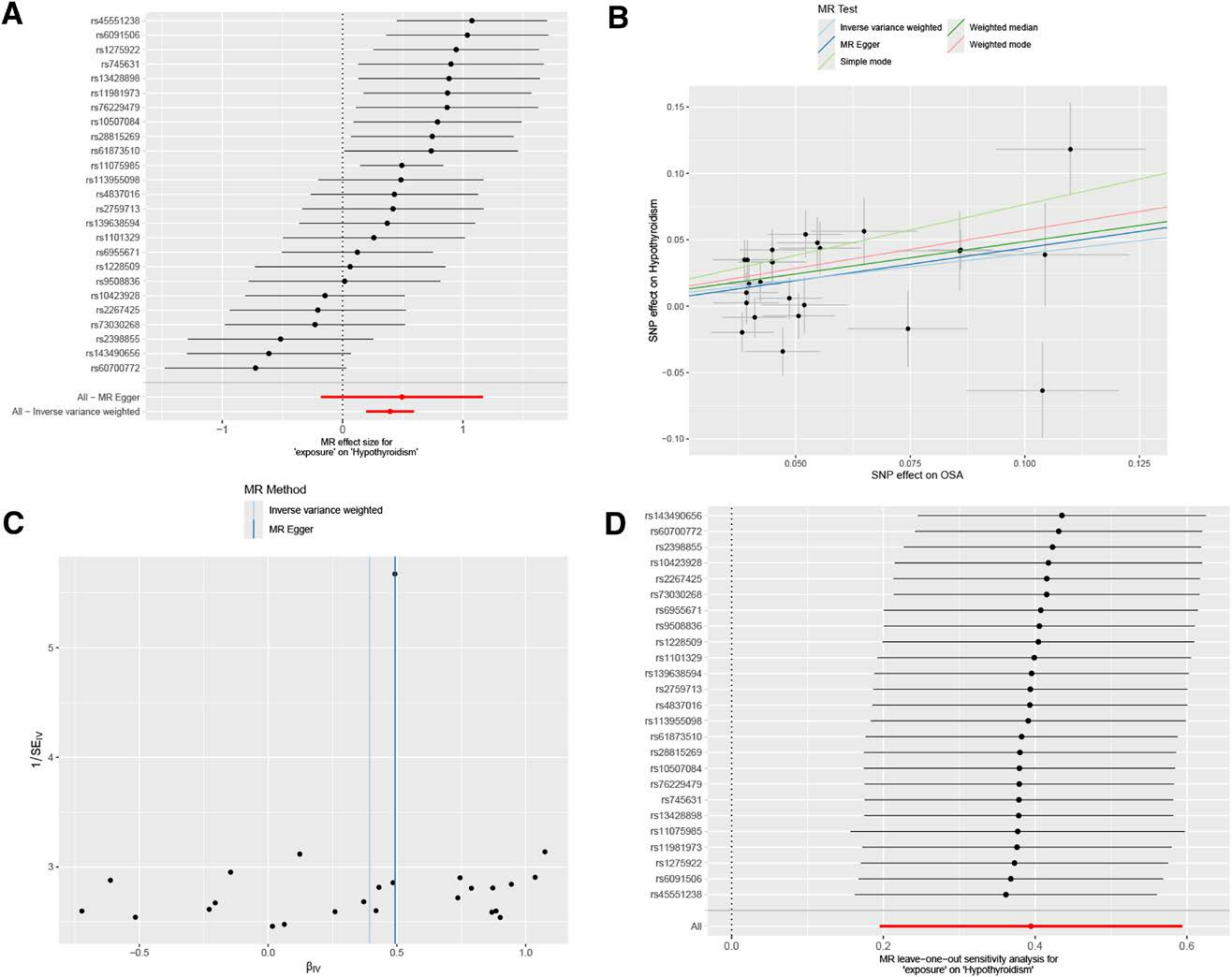
MR analysis of OSA and HT-DR. A shows the forest plot; B shows the scatter plot; Panel C shows the funnel plot; D shows the leave-one-out sensitivity analysis plot. HT-DR: hypothyroidism, drug reimbursement, OSA = obstructive sleep apnea.

The only subtype that displayed a significant positive causal relationship was HT-DR (Fig. 4), with an IVW OR of 1.484 (95% CI: 1.215–1.811, *P* < .001), suggesting a potential bidirectional causal relationship between HT-DR and OSA.

### 3.3. Sensitivity analysis

Sensitivity analyses confirmed the robustness of the causal estimates. Cochran Q tests indicated no significant heterogeneity among the instrumental variables. The MR-Egger intercept tests yielded *P*-values >.05 for most analyses, suggesting the absence of directional pleiotropy. Furthermore, leave-one-out analyses demonstrated that no single SNP substantially influenced the overall results. Detailed results of pleiotropy and heterogeneity tests are provided in Tables S1 and S2, Supplemental Digital Content, https://links.lww.com/MD/P361.

## 4. Discussion

This study uncovered subtype-specific causal relationships between HT and OSA using a bidirectional Mendelian randomization approach. The main findings include: 1. increased risk: HT-DR, HT-SA, and CIDS HT significantly increase the risk of OSA. 2. Bidirectional causality: there exists a bidirectional causal relationship between OSA and HT-DR, indicating mutual influence between the 2. 3. Protective role: Tg exerts a protective effect against OSA. These findings provide targeted screening strategies for clinical practice, suggesting priority assessment for OSA in patients with HT-DR, CIDS, and HT-SA, while also recommending thyroid function testing for OSA patients.

The subtype-specific analysis helps resolve inconsistent findings in prior studies. Previously, some MR studies have reported significant associations between HT and increased OSA risk, whereas others found weak or no significant associations.^[19–23]^ Such discrepancies could originate from the neglect of heterogeneity among HT subtypes, a critical issue addressed by our stratified approach. Moreover, despite previous reports indicating no substantial impact of OSA on thyroid function,^[24]^ our study suggests more complex, bidirectional physiological interactions.

HT may influence the occurrence of OSA through various mechanisms,^[11]^ including airway narrowing due to the deposition of glycosaminoglycans in upper airway tissues, resulting in mucosal thickening and reduced airway caliber^[25]^; alteration of upper airway muscle tone and function, predisposing to airway collapsibility during sleep^[21,26–28]^; abnormal regulation of the respiratory centers leading to decreased ventilatory responsiveness to hypoxia and hypercapnia; and HT-induced weight gain,^[29]^ which further exacerbates upper airway obstruction and promotes systemic low-grade inflammation. The differential effects of HT subtypes on OSA may be explained by their distinct pathological mechanisms. In HT-DR, patients often experience chronic HT requiring long-term thyroid hormone replacement therapy. Chronic HT is associated with generalized soft tissue edema and reduced upper airway neuromuscular tone, both of which may contribute to increased airway collapsibility.^[21,30,31]^ These effects may be exacerbated under conditions of intermittent hypoxia and sleep fragmentation typical of OSA, further impairing upper airway patency.^[32]^ In HT-SA, autoimmune-mediated destruction of the thyroid gland is often accompanied by systemic inflammatory responses, which may not only impair thyroid hormone synthesis but also contribute to inflammation and fibrosis in the upper airway. These immune-related changes can reduce upper airway caliber and stability during sleep, thereby increasing the risk of airway obstruction.^[33,34]^ Similar chronic inflammatory and mitochondrial‐driven lncRNA alterations have also been described in laryngeal squamous cell carcinoma.^[35]^ In CIDS, early-life iodine deficiency leads to congenital HT, which may disrupt the normal thyroid hormone–mediated development of the upper airway and neuromuscular system.^[36]^ Individuals with CIDS often present with features such as macroglossia, hypotonia, and craniofacial growth abnormalities, all of which are recognized risk factors for upper airway obstruction during sleep. In addition, impaired central ventilatory regulation and a tendency toward metabolic dysregulation (e.g., increased adiposity) may further exacerbate airway collapsibility. These developmental and physiological alterations may persist into adolescence and adulthood, contributing to an increased intrinsic vulnerability to obstructive sleep apnea.

The bidirectional causal relationship between OSA and HT-DR may be attributed to the chronic physiological stress imposed by OSA on the endocrine system. Intermittent hypoxia and sleep fragmentation (hallmarks of OSA) activate the hypothalamic–pituitary–adrenal axis, leading to sustained elevations in cortisol levels and disruption of normal TSH secretion rhythms.^[37]^ Chronic activation of the hypothalamic–pituitary–adrenal axis suppresses the hypothalamic–pituitary–thyroid axis, reducing TSH sensitivity and impairing peripheral conversion of thyroxine (T4) to triiodothyronine (T3). Furthermore, systemic metabolic dysregulation induced by OSA,^[38]^ including insulin resistance and dyslipidemia, can compromise thyroid hormone synthesis, while chronic low-grade inflammation (characterized by elevated interleukin-6, tumor necrosis factor-alpha, and C-reactive protein) may directly damage thyroid tissue and inhibit deiodinase activity.^[39]^ These pathophysiological changes collectively exacerbate thyroid dysfunction, particularly in individuals already predisposed to HT-DR, leading to greater disease severity or increased dependence on exogenous hormone replacement. Our findings are consistent with prior hypotheses suggesting that sleep disturbances may indirectly influence thyroid function via metabolic, inflammatory, and neuroendocrine pathways.^[40–42]^

In addition to the above findings, this study identified a significant protective causal effect of genetically predicted Tg levels against OSA risk. Tg is a precursor glycoprotein essential for the synthesis and storage of thyroid hormones. Higher Tg levels may reflect more adequate thyroid hormone reserves or more stable thyroidal homeostasis, which can help buffer against the metabolic and inflammatory perturbations associated with OSA. Additionally, Tg may have intrinsic immunomodulatory properties,^[43]^ potentially mitigating the systemic inflammation that exacerbates OSA pathophysiology. Notably, although thyroid hormones such as T3 and T4 are key regulators of metabolism and ventilatory control, we were unable to perform reliable MR analyses for T3 and T4 due to the lack of high-quality GWAS summary statistics for these biomarkers. Future studies incorporating larger GWAS datasets on T3 and T4 will be crucial for a more comprehensive understanding of the thyroid–OSA axis.

A primary strength of this study lies in its bidirectional MR design, which mitigates confounding biases. The innovative subtype analysis explains the inconsistencies in prior research results, and the multi-method verification ensures the reliability of the findings. Limitations include that the data used in the study primarily derive from individuals of European ancestry, which may affect the generalizability of the results and limit applicability to other ethnic or demographic groups. Although the MR design aims to reduce confounding biases, it cannot fully eliminate the potential impact of environmental factors on the results, which could confound causal inferences. Additionally, the lack of sufficient clinical information precludes subgroup analyses of OSA severity, which may limit deeper understanding and personalized interventions for different subtypes. Furthermore, some subtypes had a limited number of SNPs, potentially affecting the statistical power of the study and compromising the robustness of the results. While this study provides valuable insights through bidirectional Mendelian randomization, the aforementioned limitations require addressing in future research to further validate and expand upon these findings.

## 5. Conclusion

This study reveals a subtype-specific bidirectional causal relationship between HT and OSA, particularly highlighting the interaction pattern between HT-DR and OSA, as well as the protective role of Tg. These findings provide a theoretical basis for personalized risk assessments and treatment optimization in clinical practice, emphasizing the importance of multidisciplinary comprehensive management. Future research should focus on cross-ethnic validation, in-depth exploration of molecular mechanisms, and the development of treatment strategies based on thyroglobulin.

## Author contributions

**Conceptualization:** Hao Zhang, Zhimin Wu, Guodong Yu, Liang Chen, Yifei Ma, Yi Chen.

**Data curation:** Zhimin Wu, Qiu Chen, Liang Chen.

**Formal analysis:** Zhimin Wu.

**Funding acquisition:** Hao Zhang, Zhimin Wu, Guodong Yu, Liang Chen, Yifei Ma, Yi Chen.

**Investigation:** Yifei Ma.

**Methodology:** Hao Zhang, Zhimin Wu, Yi Chen.

**Project administration:** Zhimin Wu, Guodong Yu.

**Resources:** Hao Zhang, Zhimin Wu, Yifei Ma.

**Software:** Zhimin Wu, Qiu Chen, Guodong Yu, Yi Chen.

**Supervision:** Guodong Yu, Liang Chen.

**Validation:** Zhimin Wu, Guodong Yu, Yifei Ma, Yi Chen.

**Visualization:** Hao Zhang, Zhimin Wu, Guodong Yu, Liang Chen.

**Writing – original draft:** Hao Zhang, Zhimin Wu, Qiu Chen, Yi Chen.

**Writing – review & editing:** Hao Zhang, Zhimin Wu, Guodong Yu, Yifei Ma.

## Supplementary Material


