# Supplementary material for: Subtype-specific causal effects of hypothyroidism on obstructive sleep apnea: A bidirectional Mendelian randomization study
Source: Medicine (Baltimore). 2025 Jul 4;104(27):e43266. doi: 10.1097/MD.0000000000043266 (PMC12237354; doi:10.1097/MD.0000000000043266)
Supplement: Supplementary file 1 [file medi-104-e43266-s001.docx]

| **Supplementary Table 1. Pleiotropy Test Results** | | | | |
| --- | --- | --- | --- | --- |
| **outcome** | **exposure** | **egger_intercept** | **se** | **pval** |
| Hypothyroidism, strict autoimmune | OSA | 0.042 | 0.101 | 0.684 |
| Hypothyroidism, drug reimbursement | OSA | -0.005 | 0.018 | 0.766 |
| Hypothyroidism due to medicaments and other exogenous substances | OSA | -0.009 | 0.063 | 0.881 |
| Congenital iodine-deficiency syndrome/hypothyroidism | OSA | 0.033 | 0.038 | 0.397 |
| Postinfectious hypothyroidism | OSA | 0.020 | 0.083 | 0.809 |
| TRH | OSA | 0.004 | 0.008 | 0.639 |
| TSH | OSA | 0.001 | 0.025 | 0.975 |
| Thyroglobulin | OSA | 0.003 | 0.023 | 0.906 |
| OSA | Hypothyroidism, drug reimbursement | 0.001 | 0.006 | 0.918 |
| OSA | Hypothyroidism, strict autoimmune | 0.002 | 0.002 | 0.238 |
| OSA | Postinfectious hypothyroidism | 0.021 | 0.016 | 0.327 |
| OSA | Hypothyroidism due to medicaments and other exogenous substances | -0.005 | 0.005 | 0.309 |
| OSA | Congenital iodine-deficiency syndrome/hypothyroidism | -0.038 | 0.031 | 0.441 |
| OSA | TRH | 0.004 | 0.008 | 0.639 |
| OSA | TSH | 0.013 | 0.012 | 0.298 |
| OSA | Thyroglobulin | 0.005 | 0.008 | 0.549 |

| **Supplementary Table 2. Heterogeneity Test Results** | | | | | |
| --- | --- | --- | --- | --- | --- |
| **outcome** | **exposure** | **method** | **Q** | **Q_df** | **Q_pval** |
| Hypothyroidism, strict autoimmune | OSA | MR Egger | 18.484 | 23 | 0.731 |
| Hypothyroidism, strict autoimmune | OSA | Inverse variance weighted | 18.655 | 24 | 0.771 |
| Hypothyroidism, drug reimbursement | OSA | MR Egger | 52.706 | 23 | 0.418 |
| Hypothyroidism, drug reimbursement | OSA | Inverse variance weighted | 52.914 | 24 | 0.452 |
| Hypothyroidism due to medicaments and other exogenous substances | OSA | MR Egger | 16.698 | 23 | 0.824 |
| Hypothyroidism due to medicaments and other exogenous substances | OSA | Inverse variance weighted | 16.721 | 24 | 0.861 |
| Congenital iodine-deficiency syndrome/hypothyroidism | OSA | MR Egger | 26.291 | 23 | 0.287 |
| Congenital iodine-deficiency syndrome/hypothyroidism | OSA | Inverse variance weighted | 27.142 | 24 | 0.298 |
| Postinfectious hypothyroidism | OSA | MR Egger | 21.358 | 23 | 0.559 |
| Postinfectious hypothyroidism | OSA | Inverse variance weighted | 21.418 | 24 | 0.614 |
| TRH | OSA | MR Egger | 6.827 | 13 | 0.911 |
| TRH | OSA | Inverse variance weighted | 7.058 | 14 | 0.932 |
| TSH | OSA | MR Egger | 4.750 | 17 | 0.998 |
| TSH | OSA | Inverse variance weighted | 4.751 | 18 | 0.999 |
| Thyroglobulin | OSA | MR Egger | 12.456 | 22 | 0.947 |
| Thyroglobulin | OSA | Inverse variance weighted | 12.471 | 23 | 0.963 |
| OSA | Hypothyroidism, drug reimbursement | MR Egger | 65.043 | 25 | 0.217 |
| OSA | Hypothyroidism, drug reimbursement | Inverse variance weighted | 65.071 | 26 | 0.301 |
| OSA | Hypothyroidism, strict autoimmune | MR Egger | 307.793 | 158 | 0.211 |
| OSA | Hypothyroidism, strict autoimmune | Inverse variance weighted | 310.523 | 159 | 0.247 |
| OSA | Postinfectious hypothyroidism | MR Egger | 1.227 | 2 | 0.541 |
| OSA | Postinfectious hypothyroidism | Inverse variance weighted | 2.886 | 3 | 0.411 |
| OSA | Hypothyroidism due to medicaments and other exogenous substances | MR Egger | 67.414 | 39 | 0.314 |
| OSA | Hypothyroidism due to medicaments and other exogenous substances | Inverse variance weighted | 69.255 | 40 | 0.307 |
| OSA | Congenital iodine-deficiency syndrome/hypothyroidism | MR Egger | 0.965 | 1 | 0.326 |
| OSA | Congenital iodine-deficiency syndrome/hypothyroidism | Inverse variance weighted | 2.415 | 2 | 0.299 |
| OSA | TRH | MR Egger | 6.827 | 13 | 0.911 |
| OSA | TRH | Inverse variance weighted | 7.058 | 14 | 0.932 |
| OSA | TSH | MR Egger | 11.264 | 8 | 0.187 |
| OSA | TSH | Inverse variance weighted | 13.007 | 9 | 0.162 |
| OSA | Thyroglobulin | MR Egger | 8.285 | 9 | 0.506 |
| OSA | Thyroglobulin | Inverse variance weighted | 8.673 | 10 | 0.563 |
